# First Reported Case of *Candida dubliniensis* Endocarditis Related to Implantable Cardioverter-Defibrillator

**DOI:** 10.1155/2020/6032873

**Published:** 2020-01-16

**Authors:** Nooraldin Merza, John Lung, Taryn B. Bainum, Assad Mohammedzein, Shanna James, Mazin Saadaldin, Tarek Naguib

**Affiliations:** ^1^Department of Internal Medicine, Texas Tech University Health Sciences Center, Amarillo, TX, USA; ^2^School of Medicine, Texas Tech University Health Sciences Center, Amarillo, TX, USA; ^3^Department of Pharmacy Practice, Texas Tech University Health Sciences Center Jerry H. Hodge School of Pharmacy, Amarillo, TX, USA

## Abstract

A 36-year-old male presented to the ED with acute chronic hyponatremia found on routine weekly lab work with one-week history of generalized weakness, confusion, nausea/vomiting, and diarrhea. The patient has nonischemic cardiomyopathy of unknown etiology diagnosed in his teens with an AICD device placed 8 years ago and receiving milrinone infusion 3 years ago via peripherally inserted central catheter (PICC) line. Two sets of blood cultures grew *Candida dubliniensis*. The patient was started on micafungin and the PICC line was removed and replaced with a central line. A transthoracic echocardiogram (TEE) showed findings consistent with AICD lead involvement. The patient was continued on treatment for fungal infective endocarditis and transferred to another hospital where he had successful AICD lead extraction. Blood cultures upon transfer back to our facility were positive for methicillin-sensitive *Staphylococcus aureus* (MSSA). This bacteremia was thought to be secondary to right-sided internal jugular (IJ) central line and resolved with line removal and initiation of intravenous (IV) cefazolin. The patient was discharged on IV cefazolin and IV micafungin. He had a LifeVest® until completion of his antibiotic course and a new AICD was placed.

## 1. Introduction

Automatic implantable cardioverter-defibrillator (AICD) devices are used for a wide variety of cardiac conditions including secondary prevention of sudden cardiac death for patients with a previous cardiac arrest who have sustained ventricular tachycardia, coronary artery disease, nonischemic dilated cardiomyopathy, hypertrophic cardiomyopathy, and genetic arrhythmia syndromes. AICDs can also be used for the primary prevention of sudden cardiac death. [1].

Complications of AICD implantation include infections that may include vegetations on the leads or valves [2, 3]. Estimates of the rate of infection postimplantation range from 0.13% [4] to 19.9% [5]. Pocket infections related to AICD devices usually involve staphylococci and occur within weeks of implantation.

Systemic AICD device infections were less common, presented later, and typically involve a wider range of microbes compared to pocket infections. Current guidelines call for AICD removal with TEE demonstrating valve or lead vegetation, presence of pocket infection, or positive blood cultures with *S*. *aureus*, coagulase-negative staphylococci, *Cutibacterium*, *Candida* species, or other high-grade bacteremia without alternative source [6]. Antimicrobial treatment without device removal has a high reinfection and failure rate [7]. In most cases, the long-term harm to the patient of repeated infections and mortality outweigh the risks of immediate extraction with other approaches like device retention and antibiotic therapy having a higher risk of mortality [8, 9].

*Candida*-related ICD infection is extremely rare and there are only 23 *Candida* device-associated endocarditis cases published in the literature (Table 1) [10–13]. The majority of patients are over age 60 and had an infection with a time postimplantation ranging from less than a month to 16 years [14].

We report on a unique case of AICD-related fungemia complicated by simultaneous use of a chronic milrinone infusion. We describe the rare infection, the path to diagnosis, treatment, and postoperative complications observed prior to discharge.

We report the first case of *Candida dubliniensis* as a source of ICD fungemia in the English language literature. *Candida dubliniensis* is an opportunistic pathogen that was originally isolated from AIDS patients, but since then, it has been isolated from both immunocompetent and immunocompromised individuals [15, 16]. Furthermore, invasive infections by *C*. *dubliniensis* have increased considerably in recent years in immunocompromised/immunosuppressed individuals [16].

## 2. Case Presentation

A 36-year old male presented to the ED with generalized weakness, confusion, and fatigue starting one week prior. The symptoms were associated with some episodes of nausea, vomiting, and loose semisolid stools. The patient reported shortness of breath that had progressed to rest over the past weeks. He denied any fever but reported some chills. He denied any chest pain, palpitations, lower extremity swelling, abdominal pain, or other complaints. The patient has routine weekly labs which showed hyponatremia (sodium = 122 mmol/dL, baseline 130 mmol/dL) so he was sent for evaluation to the emergency room by his cardiologist. The patient has a past medical history of nonischemic cardiomyopathy diagnosed at age 16. A transthoracic echocardiogram done about 9 months prior showed a left ventricular ejection fraction of less than 20% and severe concentric left ventricular hypertrophy. The patient had an automatic implantable cardioverter-defibrillator (AICD) placement 8 years prior for primary prevention of sudden cardiac death. The patient had been on a chronic milrinone infusion delivered through a peripherally inserted central catheter (PICC) line for the past 3 years. This was initiated as a bridge to transplant. However, during transplant evaluation, he was noted to have secondary pulmonary hypertension and would need a combined heart and lung transplant, and no transplant center in the state would accept his insurance for a combined transplant. Besides cardiomyopathy, he also has a history of chronic atrial fibrillation, congenital hydrocephalus with ventriculoperitoneal shunt (since the age of 2), and spinal stenosis. Of note, the patient was taking apixaban, bumetanide, magnesium oxide, allopurinol, digoxin, metolazone, eplerenone, and carvedilol.

On exam, the patient had a red, swollen left upper extremity at the site of his PICC and white nail beds on the left hand and cool extremities, but no lower extremity edema. The patient was afebrile, had an elevated heart rate of 109 beats per minute (bpm), an elevated respiratory rate of 26 breaths per minute, and SpO2 was 96% on 2 L nasal cannula. All other vitals were within normal limits.

Labs on admission are as follows: WBC: 10.9 × 10^3^/mcL; sodium: 123 mmol/L; potassium: 4.7 mmol/L; chloride: 90 mmol/L; bicarbonate: 22; BUN: 27 mg/dL; creatinine: 0.9 mg/dL; glucose: 102 mg/dL; INR: 1.56; total bilirubin: 4.12 mg/dL; alkaline phosphatase: 166 units/L; AST: 44 units/L; and ALT: 27 units/L. The patient was initially diagnosed with viral gastroenteritis and his symptoms of nausea, vomiting, and diarrhea resolved within 2 days. As the team was preparing to discharge the patient, 2 sets of blood cultures taken at admission came back positive for *Candida dubliniensis*. The patient's WBC count increased from 11.0 to 15.2 with continued stability in his vital signs other than an increase in his heart rate to 120 s bpm. A chest X-ray on admission showed no acute abnormalities, stable cardiomegaly, and an AICD in place. An electrocardiogram (EKG) showed atrial flutter. CT scan of the head showed a left VP shunt with mild ventriculomegaly of the lateral and third ventricles unchanged since 2017. No evidence of intracranial hemorrhage was found. A lumbar puncture obtained clear CSF that was sent to microbiology. The CSF cultures had no growth. The patient was started on empiric micafungin 100 mg IV daily and vancomycin.

A blood culture of the PICC line and a culture of the catheter tip was positive for *Candida dubliniensis* as determined by matrix-assisted laser desorption/ionization time-of-flight (MALDI-TOF) mass spectrometry. Minimum inhibitory concentrations (MICs) for antifungals are listed in Table 1.

The PICC line was removed and a right-sided internal jugular vein (IJV) central line was placed. Vancomycin was discontinued after one day once the culture grew yeast. A negative blood culture of *C*. *dubliniensis* was drawn 4 weeks after the first positive culture. Micafungin was continued for a total of 6 weeks after the initial positive culture.

**Figure 1:**
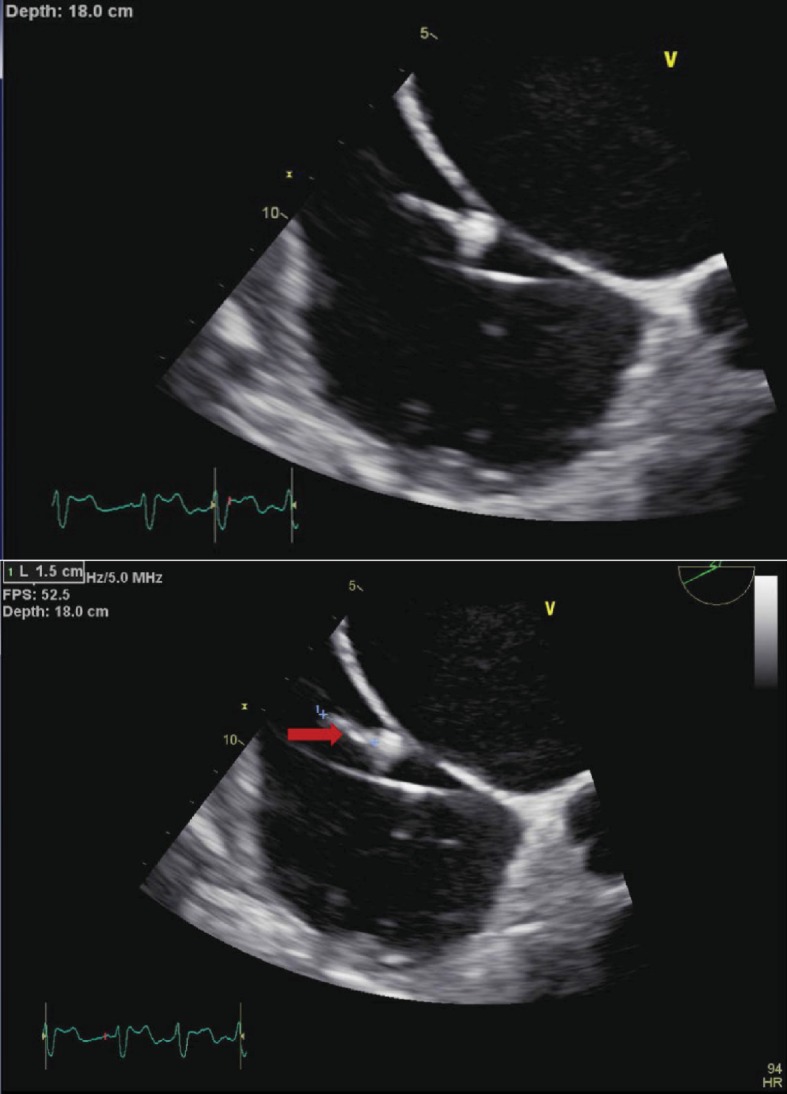
Echogenicity of one of the three leads consistent with lead vegetation measuring 0.4 × 1.3 cm. The transesophageal echocardiogram did not identify any significant valvular vegetations.

Transesophageal echocardiogram (TEE) (Figure 1) showed a new finding of echogenicity of one of the three leads consistent with lead vegetation. Consistent with a TEE done 8 months ago, there was minimal thickening of the aortic valve, mild thickening of the mitral valve with moderate to severe mitral regurgitation, and moderate to severe tricuspid regurgitation. One of the leads had a mobile echodensity measuring 0.4 × 1.3 cm, consistent with lead vegetation. No valvular vegetations were identified on TEE. The findings of the TEE were consistent with AICD fungal septicemia. An AICD lead extraction was done one week after admission at an outside facility with a temporary transcutaneous pacer placed, after which the patient returned to our inpatient facility for placement of a replacement AICD two weeks later. Blood cultures done at the outside facility were negative. Blood cultures from the right and left arms done at our facility after AICD implantation returned *Staphylococcus aureus* on 2/2 sets sensitive to cefazolin, oxacillin, tetracycline, and trimethoprim/sulfamethoxazole. The right-side IJV central line was removed and a left-side IJV central line was placed with negative blood cultures after placement. The micafungin was recommended to be continued for four weeks after new AICD placement and cefazolin was started for two weeks due to the right-sided IJV central line MSSA infection. A vascular surgeon was consulted for optimal line access with the least infectious risk. The vascular surgeon recommended a PICC line. A replacement PICC line was placed three weeks later and the patient was discharged the next day with a PICC line for milrinone, cefazolin, and micafungin infusion.

## 3. Discussion

With the increasing use of AICD devices for the secondary and primary prevention of cardiac death, clinicians should be vigilant of the possibility of infection caused by AICD placement. Risk factors that can increase infection rates include heart failure, diabetes, renal disease, immunosuppressed state, oral anticoagulation, chronic lung disease, and recent device modifications [10, 17]. Our patient had several comorbidities that increased his risk of an infection including use of oral anticoagulation, a history of heart failure, and pulmonary hypertension. His primary risk factor, however, was chronic PICC line for milrinone infusion, despite PICC being exchanged about every 6 months and the patient practicing adequate PICC care at home.

Our patient's scenario was unique because, to our knowledge, it is the first reported case of *C*. *dubliniensis*-ICD fungemia with sepsis, in the English-language literature. He denied fever which was not uncommon in candidemia. Additionally, his age was younger than the average of the other case reports which were related to AICD-*Candida* infection (see Table 2); he has nonischemic cardiomyopathy diagnosed at age 16 and has multiple comorbidities that complicate diagnosis and treatment.

*Candida dubliniensis* was first recovered postmortem from a lung specimen of a patient who expired in the United Kingdom in 1957. This organism was originally misidentified as *C*. *stellatoidea*. This species was discovered to be unique from other *Candida* species in the city of Dublin, resulting in the name *Candida dubliniensis*. This organism was found in mainly oral cavities, primarily those of HIV-infected individuals. Though this species has been found in oral cavities of healthy individuals and has been implicated infections in healthy individuals, it is rare for this organism to produce infection in immunocompetent adults. It appears that diminished T-cell activity, such as that seen in HIV, could allow overgrowth of *C*. *dubliniensis* [15, 18].

This species only accounts for a small percentage of Candida infections [15]. However, infections associated with this species have been reported (Table 3). While the table represents many of the cases in which this organism was implicated, it is by no means an exhaustive list. In reviewing these cases, it seems *C*. *dubliniensis* is most commonly found in those with some degree of immunocompromise. In particular, many reported cases have occurred in patients with liver disease and/or substance abuse. Understanding the population at risk for this infection can help clinicians identify this organism in a timely manner.

*C*. *dubliniensis* has demonstrated resistance to antifungal agents, specifically fluconazole. While these resistant strains do not account for the majority of strains, caution should be exercised when prescribing empiric therapy. In addition, fluconazole resistance is easily developed in vitro. This further highlights the need for caution and utilization of other antifungal agents if *C*. *dubliniensis* is found [15, 17].

Although our patient had some signs of sepsis including an elevated heart rate and respiratory rate, he was afebrile and appeared to have viral gastroenteritis. After a WBC count spike and *Candida* growth 2 days later, our team suspected sepsis, but did not know the source. The possibilities included the PICC line for milrinone infusion, an AICD infection, and an infection from the left VP shunt. The TEE identified a clear AICD lead infection and a CT head without contrast combined with negative CSF cultures ruled out a VP shunt infection. Additionally, the TEE detected no valvular vegetations which lowered the possibility of a PICC line-related infective endocarditis. The TEE and microbiology results made a strong case for *Candida dublinesis* infective endocarditis related to the AICD leads. The AICD device was removed at an outside facility due to patient preference with implantation of a new AICD device 5 days later. Finally, an MSSA infection related to the right-side IJV line placed in the hospital for antibiotic infusion delayed the patient's discharge further.

The patient was on IV micafungin for treatment of *Candida dubliniensis*. Recommendations from the Infectious Disease Society of America recommend the use of echinocandin (such as micafungin), amphotericin B, or amphotericin B with 5-flucytosine for treatment of *Candida* endocarditis with or without an ICD device [32, 33]. Regardless of the medication chosen, resolution of the fungemia usually occurs with ICD device removal, although one case report describes resolution of *Candida* endocarditis with medical management and no surgical intervention [34]. In our patient, treatment was continued for 4-6 weeks after ICD device removal.

### 3.1. Learning Points/Take Home Messages

- *Candida dubliniensis* is an opportunistic pathogen originally isolated from AIDS patients but can be isolated from immunocompetent individuals
- We report the first case in literature of *Candida dubliniensis*-ICD sepsis with fungemia and multiple possible sources of infection including a VP shunt and an indwelling PICC for milrinone infusion
- Clinicians should do a full sepsis workup for all patients admitted to the hospital with an ICD device, tachycardia, and tachypnea even in absence of fever and leukocytosis

## Figures and Tables

**Table 1:** MIC concentrations for *Candida dubliniensis*. Susceptibility results were not published in the report due to the lack of outcomes data for less common species including *C*. *dubliniensis*.

Antifungal	MIC (*μ*g/mL)
Amphotericin B	0.25
Caspofungin	0.06
Fluconazole	0.25
Flucytosine	0.06
Itraconazole	0.06
Ketoconazole	0.015
Voriconazole	0.008

**Table 2:** Summary of the reported cases of *Candida*-associated ICD septic fungemia.

Case	Age (yrs)	Gender	Illnesses	Device type	Length of device use before infection	Symptoms	Echo result	Culture results	Management/outcome
Davis et al. 1969	71	Male	Diabetes, obstructive uropathy, UTI, CHF	Permanent pacemaker	9 months	Fever, confusion, leukocytosis	Not reported	Blood: no growth; urine: yeast	Broad-spectrum antibacterials. Patient expired.
Cole et al. 1986	65	Male	CVA, IV catheter-related *Candida albicans* fungemia 6 months prior	Permanent pacemaker	8 years	Fever, confusion, urine/fecal incontinence	5 × 2 × 2 cm shaggy mass attached to pacer wire extending from RA to RV	Initial blood and urine cultures: no growth. Subsequent blood culture: *Candida albicans*	Broad-spectrum antibacterials followed by amphotericin B. Thoracotomy. Expired at surgery.
Wilson et al. 1993	56	Male	Heart block	Permanent pacemaker	5 years	Fever, cough, dyspnoea, leukocytosis	Multiple large RA masses prolapse into RV. Possible adherence to pacer wire. (TTE)	Blood: *Candida albicans*	Amphotericin B (2 g total). Right atriotomy and pulmonary arteriotomy. Removed leads and fungus ball from left main PA. Recovered, well after 2 years
Shmuely et al. 1997	75	Male	Diabetes, sick sinus syndrome	Permanent pacemaker	2 years	Blurred vision, endophthalmitis	3 cm vegetation on pacer wire below TV, within RV (TEE)	Blood: *Candida tropicalis*	Amphotericin B+5-flucytosine. Refused surgery to remove PPM. Expired with multiorgan failure
Joly et al. 1997	56	Male	Chronic bronchitis, sinus dysfunction	Permanent pacemaker	4 years: old PPM wires, 3 months: new PPM	Fever, dyspnoea	RA mass (TEE)	Initial blood culture: no growth. Blood then positive for *Candida albicans*. Wires and vegetation: *Candida albicans*	Right atriotomy: removed vegetation, wires, and PPM. Amphotericin B+5-flucytosine, then oral fluconazole × 7 months. Recovered
Cacoub et al. 1998	56	Male	Sick sinus syndrome	Permanent pacemaker	Not reported	Fever, dyspnoea	Vegetation on pacer lead	Blood and pacer lead: *S*. *epidermidis, Candida albicans*	Antibiotic. Surgical removal of PPM. Survived.
Victor et al. 1999	72	Male	Bradycardia, tachycardia syndrome	Permanent pacemaker	<1 month	Not reported	Vegetation on TV	Lead culture: *Candida glabrata*	Endovascular extraction of PPM. Expired after 2 months with active Candida endocarditis
Kurup et al. 2000	77	Male	Diabetes, coronary artery disease, sick sinus syndrome	Permanent pacemaker	5 months	Fever, dyspnoea, lethargy	TV vegetation (TTE)	Blood, vegetation: *Candida tropicalis*	Amphotericin B. Thoracotomy: vegetation on TV and PPM lead. Removed PPM and vegetations. Expired with multiorgan failure after surgery.
Roger et al. 2000	87	Male	CML, renal neoplasm, prosthetic AV	Permanent pacemaker	16 years	Fever, renal insufficiency	7 cm vegetation on pacer wire (TTE+TEE)	Blood, vegetation: *Candida albicans* and *Candida glabrata*	Fluconazole. Not a surgical candidate. Expired with fatal stroke
Brown et al. 2001	49	Male	Diabetes, coronary artery disease, CHF, ventricular tachycardia	Implanted cardioverter defibrillator	12 months	Fever, dyspnoea, cough, leukocytosis	3.5 cm vegetation on defibrillator lead (TTE)	Blood and vegetation: *Candida albicans*	Amphotericin B × 8 weeks, then fluconazole 400 mg P.O. daily. Explanted device by thoracotomy. Clinically stable 6 months later
Hindupur and Muslin 2005	63	Male	Coronary artery disease, CHF, ventricular tachycardia	Implanted cardioverter-defibrillator	10 months	Fatigue	Vegetations on atrial ICD lead (largest: 1.6 cm) (TTE+TEE)	Blood, ICD lead and pocket: *Candida albicans*	Removed generator, percutaneous extraction of ICD lead. Lead fractured, embolised with vegetation into left PA. Received fluconazole, then amphotericin B. improved, then expired with P. aeruginosabacteraemia
Ho et al. 2006	56	Male	Rheumatic heart disease, cardiomyopathy, ventricular tachycardia	Implanted cardioverter-defibrillator	12 years; generator change 1 week before	Fever, sweat, hypotension, ICD pocket dehisced	1.8 cm mobile vegetation on intracardiac lead (TEE)	Blood: *Candida parapsilosis*	Fluconazole IV × 6 weeks, then oral fluconazole 400 mg da: 1 lifelong. Explanted device. Survived
Talarmin et al. 2009	76	Male	Colorectal cancer	Permanent pacemaker	Not reported	Not reported	Not reported	Blood: *Candida parapsilosis*	Removed PPM: found vegetations on leads. Received fluconazole for 42 days. Expired secondary to abdominal surgery complications
Falcone et al. 2009	38	Male	Previous aortic valve replacement	Permanent pacemaker	3 months	Fever	Vegetations on pacer lead	Lead culture: *Candida parapsilosis*	Removed PPM. Received caspofungin for 6 weeks, then 12 weeks oral fluconazole and posaconazole. Cured at 14 months follow-up
Durante-Mangoni and Nappi 2010	19	Male	Complete heart block	Permanent pacemaker	1 year	Fever, cough, hemoptysis	Massive, mobile structure on pacer lead	Blood: *Candida albicans*	Caspofungin and fluconazole × 8 weeks, removal and replacement of ICD. Recovered.
Halawa et al. 2011	80	Male	Coronary artery disease, COPD, atrial fibrillation, complete heart block	Permanent pacemaker	12 years	Chills, confusion	0.5 × 0.5 cm mobile mass on pacer wire, fibrinous strands on TV	Blood and pacer vegetation: *Candida parapsilosis*	Amphotericin B, maintained for 3 weeks after PPM removed. PPM explantation and percutaneous lead extraction, no infection at 1 year follow-up.
Grunberg et al. 2013	62	Male	CHF, diabetes, coronary artery disease, hepatitis C infection	Implanted cardioverter-defibrillator	11 months	Fever, dyspnea on exertion, chest pressure	4 cm mass on ICD lead	Blood: *Candida albicans*	Fluconazole IV, ICD removal. Plan 6 weeks of fluconazole before ICD reimplantation.
Tascini et al. 2013	75	Female	Symptomatic bradycardia	Permanent pacemaker	6 years	Fever × 2 weeks	2 cm vegetation adherent to the atrial lead of the bicameral PM	Blood: *Candida albicans*	IV fluconazole × 10 days, then IV micafungin × 75 days. Survived.
Rivera et al. 2014	60	Female	HF with reduced EF, sarcoidosis, and diabetes	Implanted cardioverter defibrillator	2 years, 2 months	Fevers, chills, sweats, cough	Mobile 2.09 cm × 4.49 cm mass associated with ICD wire	Blood: *Candida albicans*	Removed ICD. Micafungin × 2 weeks, then fluconazole × 6 weeks. Survived.
Bandyopadhyay et al. 2015	86	Male	Diabetes	Permanent pacemaker	3 years	Weakness, fever	Vegetation on the pacemaker electrode in right atrium and ventricle	Blood: *Candida tropicalis*	IV caspofungin × 10 days, then IV fluconazole ×15 days, then oral fluconazole × 2 months. Survived.
Glavis-Bloom et al. 2015	70	Female	CHF, diabetes, chronic kidney disease	Implanted cardioverter-defibrillator	13 months	Fever, nausea, vomiting, fatigue	Multiple ICD lead masses and 0.7 cm mobile aortic valve mass	Blood and urine: *Candida glabrata*	Caspofungin IV × 3 days, then micafungin IV and flucytosine IV. Expired with multiorgan failure 1 month later.
Jain et al. 2018	60	Female	Diabetes, ischemic cardiomyopathy, pancytopenia	Implanted cardioverter-defibrillator	Not reported	Fever, altered mental status	Mass attached to the tricuspid valve	Blood: *Candida parapsilosis*	Amphotericin B, removed ICD and leads, removed tricuspid valve vegetations. Survived.
Jones et al. 2018	25	Female	Obesity, hypertension, diabetes, nonischemic cardiomyopathy	Implanted cardioverter-defibrillator	2 years, 9 months	Not reported	Large vegetation above the tricuspid valve, 2.1 cm × 1.6 cm echodensity within the right atrium	Blood: *Candida albicans*	AngioVac aspiration and laser sheath extraction of ICD lead, suppressive fluconazole. Survived.

UTI: urinary tract infection; CHF: congestive heart failure; CVA: cerebral vascular accident; PPM: permanent pacemaker; TEE: transesophageal echocardiogram; HF: heart failure; EF: ejection fraction.

**Table 3:** Case reports of infection with *Candida dubliniensis*.

Case report	Patient age and sex	Patient comorbidities	Site of infection
[19]	74, M	Chronic lymphocytic leukemia, COPD, CAD, hypertension	Blood
	30, F	End-stage liver disease, alcohol and drug abuse	Blood
	39, M	End-stage liver disease, lymphadenopathy, diabetes mellitus	Blood
	37, F	Intravenous drug use, chronic DVTs, valvular heart disease	Blood
[20]	46, F	End-stage liver disease, liver transplant	Blood, abdominal wound, tracheal aspirate
[21]	30, M	Intravenous drug user, hepatitis C	Blood
[22]	71, M	End-stage liver cirrhosis	Sputum
[23]	38, M	No past medical history	Endophthalmitis
[24]	53, M	Alcohol abuse	Fungal bezoar encapsulating a calculus in right upper kidney
[25]	62, F	Rheumatic heart disease, mitral valve replacement, thyroid papillary carcinoma, congestive heart failure	Central venous catheter and blood
	71, M	Bladder cancer	Central venous catheter and blood
[26]	31, M	Intravenous drug use	Blood, sputum, endophthalmitis
[27]	75, F	Laryngeal cancer, tuberculosis	Pneumonia
[28]	49, M	Hepatitis C, cirrhosis, substance use disorder, recent exposure to IV antibiotics	Meningitis
[29]	56, M	Diabetes mellitus type 2	Right hand abscess
[30]	59, M	COPD, diabetes mellitus type 2, ulcerative colitis	Pneumonia
[31]	45, F	None reported	Keratitis
